# Anxiety and subjective assessment of cognitive functions after COVID-19.

**DOI:** 10.1192/j.eurpsy.2024.556

**Published:** 2024-08-27

**Authors:** T. I. Medvedeva, O. M. Boyko, S. N. Enikolopov, O. U. Vorontsova

**Affiliations:** Clinical psychology, Federal Stare Budgetary Scientific Institution “Mental Health Research Center”, Moscow, Russian Federation

## Abstract

**Introduction:**

Wide circulation of the COVID-19 has led to the high occurrence of a longcovid in which the complaints of violations of cognitive functions and affective disorders often occur.

**Objectives:**

The aim of this study was to assess the relation of anxiety and subjective appraisal of the states of cognitive functions.

**Methods:**

The data of 1233 respondents of internet-research who were divided into the four groups according to their COVID-19 status and the level of anxiety. Group 1 (didn’t have COVID before, low level of anxiety) – 689 people (mean age 40,6), group 2 (didn’t have COVID before, High level of anxiety) – 364 people (mean age 39,8), group 3 (had been ill COVID-19, low level of anxiety) – 102 people (mean age 41,2), group 4 (had been ill COVID-19, High level of anxiety) -130 people (mean age 35,5). Methods include the questions about the states of their cognitive functions (attention, memory, working capacity), a question about COVID-19 status. There are the results of comparing the groups that was carried out using the Kruskal-Wallis test. A pairwise comparison was carried out using the Mann-Whitney test for two groups of people who were not ill; two groups who were ill; two groups with a low level of anxiety; two groups with a high level of anxiety. To correct multiple comparisons, the adjusted significance level calculated by the formula (p = 1 - 0,951^1/n^) was used, which was p=0,017 for 4 pairwise comparisons.

**Results:**

Results are shown in table.

**Table:** 

	Group 1	Group 2	Group 3	Group 4
Trouble remembering things	0,50	0,99	0,77	1,30
	(*2)	(*1,*4)	(*1,*4)	(*2,*3)
Feeling low in energy or slowed down	0,74	1,77	1,23	2,34
	(*2,*3)	(*1,*4)	(*1,*4)	(*2,*3)
Having to do things very slowly to insure correctness	0,27	0,88	0,31	1,00
	(*2)	(*1)	(*4)	(*3)
Difficulty making decisions	0,63	1,63	0,82	1,67
	(*2)	(*1)	(*4)	(*3)
Your mind going blank	0,34	1,12	0,64	1,36
	(*2,*3)	(*1)	(*1,*4)	(*3)
Trouble concentrating	0,58	1,55	0,72	1,86
	(*2)	(*1,*4)	(*4)	(*2,*3)
Feeling everything is an effort	0,43	1,47	0,56	1,81
	(*2)	(*1,*4)	(*4)	(*2,*3)

An entry in parentheses such as (2*) means that this group for this parameter statistically significant differs from group 2.

As indicated in the table, respondents with the high level of anxiety have higher levels of the subjective assessment of cognitive functions regardless of their COVID-19 status.

**Conclusions:**

A possible explanation may be the disorganizing effect of anxiety on the cognitive functions. When combined with possible organic disorders caused by the transferred COVID-19, the most marked indicators of cognitive decline are observed. An effective rehabilitation of cognitive functions after COVID-19 requires to diagnose the level of anxiety and to seek psychological and psychiatric assistance for people with a high level of anxiety.

**Disclosure of Interest:**

None Declared

